# Subthreshold Micropulse Photocoagulation for Persistent Macular Edema Secondary to Branch Retinal Vein Occlusion including Best-Corrected Visual Acuity Greater Than 20/40

**DOI:** 10.1155/2014/251257

**Published:** 2014-09-04

**Authors:** Keiji Inagaki, Kishiko Ohkoshi, Sachiko Ohde, Gautam A. Deshpande, Nobuyuki Ebihara, Akira Murakami

**Affiliations:** ^1^Department of Ophthalmology, St. Luke's International Hospital, 9-1 Akashi-cho, Chuo-ku, Tokyo 104-8560, Japan; ^2^Department of Ophthalmology, Juntendo University Graduate School of Medicine, Hongo 2-1-1, Bunkyo-ku, Tokyo 113-8421, Japan; ^3^Center for Clinical Epidemiology, St. Luke's Life Science Institute, 10-1 Akashi-cho, Chuo-ku, Tokyo 104-0044, Japan

## Abstract

To assess the efficacy of subthreshold micropulse diode laser photocoagulation (SMDLP) for persistent macular edema secondary to branch retinal vein occlusion (BRVO), including best-corrected visual acuity (BCVA) > 20/40, thirty-two patients (32 eyes) with macular edema secondary to BRVO were treated by SMDLP. After disease onset, all patients had been followed for at least 6 months prior to treatment. Baseline Snellen visual acuity was used to categorize the eyes as BCVA ≤ 20/40 (Group I) or BCVA > 20/40 (Group II). Main outcome measures were reduction in central macular thickness (CMT) in optical coherence tomography (OCT) and BCVA at 6 months. In the total subject-pool at 6 months, BCVA had not changed significantly but CMT was significantly reduced. Group I exhibited no significant change in CMT at 3 months but exhibited significant reductions at 6 and 12 months. Group II exhibited a marginally significant reduction in CMT at 3 months and a significant reduction at 6 months. In patients with persistent macular edema secondary to BRVO, SMDLP appears to control macular edema with minimal retinal damage. Our findings suggest that SMDLP is an effective treatment method for macular edema in BRVO patients with BCVA > 20/40.

## 1. Introduction

Macular edema is one of the most important causes of impaired vision in patients with branch retinal vein occlusion (BRVO), and, in 1984, the Branch Vein Occlusion Study (BVOS) group demonstrated the efficacy of grid laser photocoagulation to improve visual acuity in patients with macular edema secondary to BRVO [1]. Recently, Campochiaro et al. [2] revealed that antivascular endothelial growth factor (VEGF) therapy for macular edema due to BRVO in patients with best-corrected visual acuity (BCVA) of ≤20/40 yields greater BCVA improvement than conventional laser therapy. Based on previous reports [1–4], most patients with BCVA of > 20/40 are observed without any intervention until visual acuity drops to ≤20/40. However, we have observed numerous chronic-stage patients in whom some degree of macula edema persists long after hemorrhage resolution, though BCVA is maintained at >20/40. These patients often complain of reduced vision or metamorphopsia. Conventional laser therapy [1, 5] inevitably results in retinal scarring [6–8] and reduced macular sensitivity [9] in some patients. Thus, in patients with good BCVA (such as >20/40), conventional grid laser therapy seems overly invasive, and thus undesirable.

Subthreshold micropulse diode laser photocoagulation (SMDLP) is a less invasive treatment than conventional grid laser therapy, designed to produce lesions on the retinal pigment epithelium (RPE) while having minimal effect on the sensory retina and choroid [10, 11]. Pulsed laser ablation is frequently performed during the procedure, without damaging the photoreceptor layer [12]. This treatment has been demonstrated to improve or resolve macular edema without any laser scarring [13].

Several reports have been published on the efficacy of SMDLP for diabetic macular edema and/or macular edema secondary to BRVO [14–23]. In 2006, Parodi et al. first reported a clinical investigation of SMDLP for macular edema secondary to BRVO with BCVA ≤ 20/40 [22]. Since then, only 2 clinical studies from the same study-group have demonstrated the efficacy of this method for macular edema secondary to BRVO with BCVA ≤ 20/40 [22, 23].

A reliably efficacious treatment for persistent macular edema secondary to BRVO in patients with BCVA > 20/40 has not yet been established, and this is the first study of SMDLP for macular edema secondary to BRVO in Japanese patients. The aim of this pilot study was to investigate the efficacy of SMDLP for persistent mild or moderate macular edema secondary to BRVO, including patients with BCVA of >20/40.

## 2. Materials and Methods

The study reported herein was a single-center, retrospective, and nonrandomized, interventional case series. Thirty-two consecutive patients (32 eyes) with macular edema secondary to BRVO were recruited for SMDLP between the 6th of October, 2003, and the 24th of May, 2012. We obtained approval from the Research Ethics Committee of St. Luke's International Hospital prior to study initiation, and the study followed the tenets of the Declaration of Helsinki.

Prior to treatment, all patients had been followed up for at least 6 months after disease onset, and their macular edema had been confirmed as persistent. Eligibility criteria included BRVO (ischemic or nonischemic) with persistent mild or moderate macular edema with a central macular thickness (CMT) of <600 *μ*m, as determined by optical coherence tomography (OCT). Macular edemas exhibiting CMT values of ≥600 were excluded, because laser energy is not sufficient for treating severe macular edema [20]. All patients had reduced vision or metamorphopsia, due to persistent or increasing macular edema for at least 2 visits after the resolution of dense hemorrhage. Patients who complained of metamorphopsia due to macular edema and had a BCVA of >20/40 or refused additional pharmacotherapy (steroid or anti-VEGF therapy) were also included.

Baseline BCVA ranged from 20/222 to 20/20 on Snellen equivalency (0.35 ± 0.29; mean ± SD). Fluorescein angiography was performed to confirm diffuse dye leakage and rule out focal capillary nonperfusion at recruitment. Subfoveal hard exudate and epiretinal membrane formation were excluded, as were patients with macular hemorrhage precluding sufficient laser ablation. Other exclusion criteria included a history of cataract surgery, any other intraocular surgery within 3 months prior to the study, and previous therapy for macular edema (including subtenon injection of triamcinolone, intravitreal injection of any drug, or macular grid laser photocoagulation) within 6 months prior to the study. During the study period, 34 eyes of patients with macular edema due to BRVO received SMDLP. Of these, 2 eyes with conventional grid photocoagulation, after 1 month of operation, were excluded. Thus, a total of 32 eyes were included in the analysis. All patients in this study had their BCVA, CMT, and TMV evaluated at all time-points up to the study endpoint (baseline, 1 month, 3 months, 6 months, and 12 months).

After providing informed consent, each patient underwent SMDLP. All treatments were performed by the same surgeon, with 33 years of surgical experience in ophthalmology. An 810-nm diode laser photocoagulation device (Iris Medical OcuLight Slx, Iridex Corporation, Mountain View, CA) was used, in “micropulse” operating mode. Laser light was delivered to the involved macular region inside arcade vessels via a slit lamp adapter through a three-mirror contact lens. Laser power for subthreshold treatment was determined for each patient by creating a threshold burn with the lowest energy required to make a visible “test burn” in an appropriate area outside the vascular arcade without retinal edema. The laser was subsequently used at 60%–90% of that energy level in micropulse mode and applied to confluent spots up to 500 *μ*m from the center of the fovea. The test burn was created with continuous wave laser energy (100% duty cycle) for 0.1 s at a diameter of 200 *μ*m. In 13 eyes, laser spots were applied with the 15% duty cycle micropulse mode at 200% of threshold energy, 878.46 ± 215.05 mW (mean ± SD) (750 mW to 1500 mW) for 0.3 s, resulting in the delivery of 90% of the threshold energy.In 19 eyes, laser spots were applied with the 15% duty cycle micropulse mode at 200% of threshold energy, 933.68 ± 417.81 mW (mean ± SD) (360 mW to 2000 mW) for 0.2 s, resulting in the delivery of 60% of the threshold energy.

BCVA and macular parameters were examined at enrollment and at 1, 3, 6, and 12 months after treatment. BCVA was determined with the Snellen chart, and logarithm of the minimum angle of resolution (log MAR) values were calculated. CMT and total macular volume (TMV) were measured using either a Stratus OCT 3000 or a Cirrus HD-OCT (Zeiss Humphrey Instruments, Dublin, CA), with TMV measured in the “fast macular” scan mode (between the 6th of October 2003 and the 23rd of October 2009) or “cube” scan mode (between the 26th of October 2009 and the 22nd of May 2013). According to a report by Abedi et al. [24], CMT values equivalent to those determined by an OCT 3000 instrument can be calculated by subtracting 60 from CMT values determined by a Cirrus HD-OCT instrument. Patients were followed at monthly intervals for at least 3 months, without any additional treatment. Subsequently, additional treatment was limited to SMDLP, which was provided as needed for persistent macular edema and/or reduced BCVA.

Parodi et al. [22] reported that the endpoint of the effect of SMDLP was evaluated at 6 months. Thus, the main outcome measures in this study were decrease in CMT on OCT and BCVA at 6 months. Statistical analyses were performed using the Wilcoxon signed rank test, Friedman test, and Mann-Whitney *U* test to evaluate these outcomes, while the Friedman test was used to evaluate trends in parameters over time. The SPSS software package (Chicago, Illinois, USA) was used for all statistical analyses, and *P* < 0.05 was used to indicate statistical significance.

## 3. Results

### 3.1. Demographic Data and Baseline Characteristics

Thirty-two patients (32 eyes, 23 men and 9 women; mean age 66.9 ± 9.74 years; age range 49–88 years) with persistent macular edema secondary to BRVO were enrolled in this study and underwent SMDLP. BRVO onset ranged from 6 to 156 months prior to treatment, with a mean of 34.6 ± 37.5 months. Previous treatments at least 6 months prior to enrollment included subtenon triamcinolone injection in 7/32 eyes (21.9%), intravitreal triamcinolone injection in 2/32 eyes (6.3%), intravitreal bevacizumab injection in 2/32 eyes (6.3%), macular grid laser photocoagulation in 6/32 eyes (18.8%), and vitrectomy in 4/32 eyes (12.5%). Overall, 11/32 eyes (34.4%) had undergone treatment with steroid or anti-VEGF therapy.

BCVA was used to categorize eyes into 2 groups: Group I (15/32 eyes, 46.9%) with BCVA ≤ 20/40 and Group II (17/32 eyes, 53.1%) with BCVA > 20/40. Macular BRVO was present in 14 of the 32 eyes (43.8%) included in this study. Ischemic type, defined by detection of retinal capillary nonperfusion ≥5 disc diameters, was apparent in 13/32 eyes (40.6%). Nonischemic type was apparent in 19/32 eyes (59.4%). Preoperative CMT ranged from 181 *μ*m to 573 *μ*m (mean 390.2 ± 94.9 *μ*m). Characteristics of the patients in the two groups are shown in Table 1.

### 3.2. Further Treatment

All patients completed 3 months of follow-up, after which additional SMDLP was performed in 11 eyes (31.3%; 8 in Group I, 3 in Group II) within the subsequent 12 months to treat persistent macular edema. A summary of the results in each group, including those who did and did not receive additional SMDLP administration is shown in Table 2.

### 3.3. Macular Parameters and OCT Findings at 6 Months

**Figure 1:**
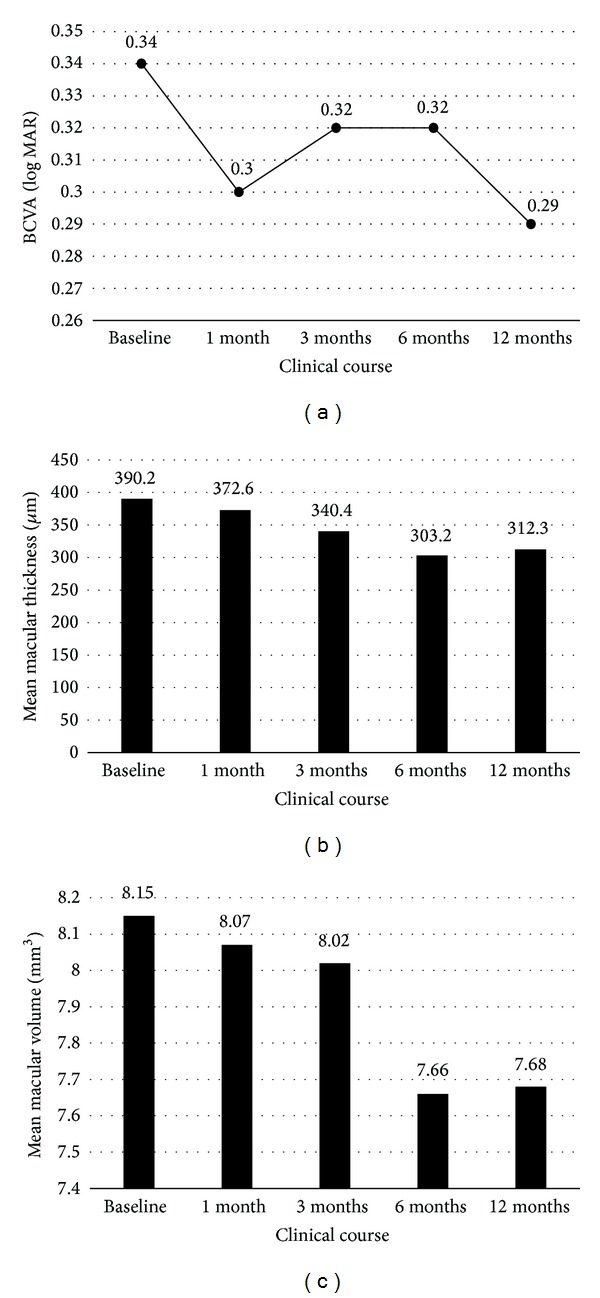
Changes in postoperative parameters: 12-month follow-up of all patients after subthreshold micropulse diode laser photocoagulation (SMDLP). (a) Changes in visual acuity (logarithm of the minimum angle of resolution) over time after SMDLP. Visual acuity showed significant improvements at 1 month and 12 months (*P* = 0.004 and 0.046 resp.). (b) Changes in macular thickness over time after SMDLP. Central macular thickness (CMT) showed a significant decrease at 3 months, and remained stable thereafter (*P* < 0.01). (c) Changes in macular volume over time after SMDLP. Macular volume showed significant reductions at 1, 6, and 12 months (*P* = 0.049, 0.002, and 0.049 resp.).

At 6 months after SMDLP, a significant reduction in CMT was evident (*P* = 0.00026), as was a significant change in TMV (*P* = 0.002) (Figures 1(a) and 1(b)). Preoperative mean CMT and TMV were 390.2 ± 94.9 *μ*m and 8.15 ± 0.95 mm^3^, respectively, while at 6 months they were 303.16 ± 108.15 *μ*m and 7.66 ± 0.61 mm^3^ (Table 3). Overall, 25/32 eyes (78.1%) showed a reduction in CMT at 6 months and CMT decreased by at least 20% in 16/32 eyes (50%). In Group I, there was a significant change in CMT at 6 months (*P* = 0.009). In Group II, there was a significant change in CMT at 6 months (*P* = 0.015) (Figure 3(a)).

### 3.4. BCVA at 6 Months

The change in BCVA (log MAR) at 6 months, from 0.34 ± 0.28 to 0.32 ± 0.34 (20/59 to 20/62 in Snellen equivalence, Figure 1) was not significant. In Group II, 16/17 eyes (94.1%) maintained a BCVA > 20/40 at 6 months (Table 4).

### 3.5. Changes in BCVA and Macular Parameters Throughout 12 Months of Follow-Up

**Figure 2:**
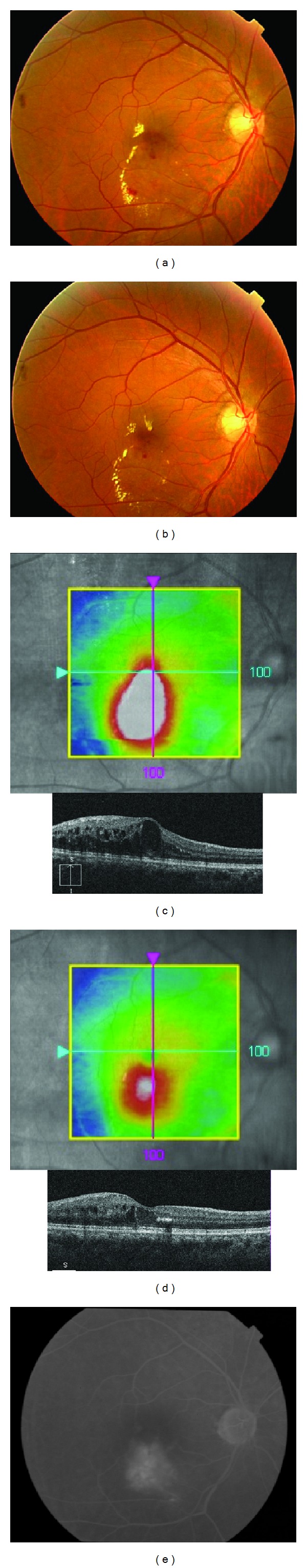
Persistent macular edema secondary to branch retinal vein occlusion with best-corrected visual acuity (BCVA) > 20/40 treated by SMDLP. (a) Fundus color photograph obtained before SMDLP, showing cystoid macular edema. (b) Fundus color photograph at 3 months after SMDLP. (c) Optical coherence tomography at baseline. (d) Optical coherence tomography at 3 months after treatment. The baseline horizontal scan shows a cystoid area at the fovea that has improved at 3 months. CMT was 525 *μ*m at baseline and 346 *μ*m at 3 months. BCVA (logarithm of the minimum angle of resolution) was 0.046 before SMDLP and 0 at 3 months. (e) Baseline fluorescein angiography revealing diffuse dye leakage in the macular area. SMDLP was applied to the area of diffuse dye leakage.

Mean BCVA was significantly improved at 1 month and at 12 months (*P* = 0.004 and *P* = 0.046 resp.) (Figure 1(a)). Mean BCVA was not significantly improved at 3 months or at 6 months (*P* = 0.214 and *P* = 0.119 resp.). CMT reductions remained significant at 3, 6, and 12 months (*P* = 0.014, 0.00026, and 0.006 resp.) (Figure 1(b)). CMT reduction was not significant at 1 month (*P* = 0.172). Changes in TMV were significant at 1, 6, and 12 months (*P* = 0.026, 0.002, and 0.049 resp.). Change in TMV was marginally significant at 3 months (*P* = 0.092) (Figure 1(c) and Figures 2(a)–2(e)).

**Figure 3:**
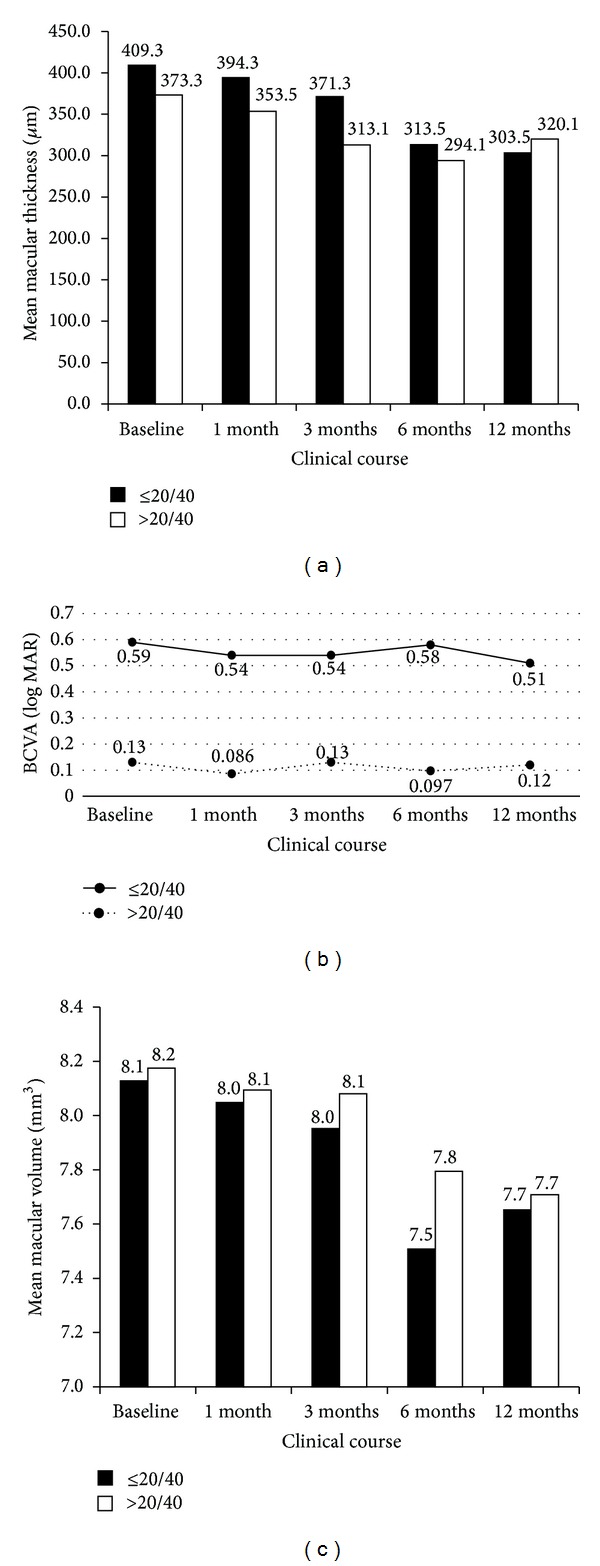
(a) Comparison of CMT in Group I (eyes with BCVA ≤ 20/40) versus Group II (eyes with BCVA > 20/40). No significant differences in CMT were evident between Group I and Group II at any time-point (baseline, 1, 3, 6, or 12 months) after the operation. In Group I, CMT showed significant reductions at 6 months and 12 months (*P* = 0.009 and 0.041 resp.). In Group II, CMT showed a significant reduction at 6 months (*P* = 0.015) and marginally significant reductions at 3 months and 12 months (*P* = 0.062 and 0.098 resp.). (b) Change in visual acuity over time after SMDLP in Groups I and II. There was no significant change in visual acuity throughout 12 months (Group I *P* = 0.129, Group II *P* = 0.245). (c) Comparison of total macular volume (TMV) in Group I (eyes with BCVA ≤ 20/40) versus Group II (eyes with BCVA > 20/40). No significant differences in TMV were evident between Group I and Group II at any time-point (baseline, 1, 3, 6, and 12 months) after the operation. In Group I, TMV showed significant reductions at 1 month and 6 months (*P* = 0.041 and 0.028 resp.). In Group II, TMV showed a significant reduction at 6 months (*P* = 0.016).

BCVA data are summarized in Table 3. BCVA was improved or maintained within 0.2 log MAR in 29/32 eyes (90.6%) at 6 months, and 30/32 eyes (93.8%) showed improvement or maintenance of BCVA at 12 months. Of the 21/32 (65.6%) eyes without additional treatment, 20/21 (95.2%) showed an improvement in BCVA of ≥0.2 log MAR or maintained it within 0.2 log MAR for 12 months. There was no significant change in BCVA in either group, at 12 months (Group I, *P* = 0.129; Group II, *P* = 0.245) (Figure 3(b)). Data on CMT, TMV, and ≥20% macular thickness reduction rate are shown in Table 3.

#### 3.5.1. Comparison of Macular Parameters in Eyes with BCVA ≤ 20/40 and Eyes with BCVA > 20/40

Clinical courses of CMT and TMV are shown in Figures 3(a) and 3(c), respectively. At no time-point (baseline, 1, 3, 6, or 12 months) did Group I or Group II show any significant difference in CMT or TMV. In Group I, CMT was significantly reduced at 6 months and 12 months (*P* = 0.009 and 0.041 resp.) but not at 1 month or 3 months (*P* = 0.281 and 0.125 resp.). In Group II, CMT was significantly reduced at 6 months (*P* = 0.015) and was marginally reduced at 3 months and 12 months (*P* = 0.062 and 0.098 resp.) and there was no significant reduction at 1 month (*P* = 0.368). In Group I, TMV was significantly reduced at 1 month and 6 months (*P* = 0.041 and 0.028 resp.) but not at 3 months or 12 months (*P* = 0.278 and 0.182 resp.). In Group II, TMV was significantly reduced at 6 months (*P* = 0.016) but not at 1 month, 3 months, or 12 months (*P* = 0.201, 0.173, and 0.208 resp.).

#### 3.5.2. Adverse Events and Macular Changes

When before and aftercolor fundus photographs were compared, no laser scars secondary to treatment were detected. Likewise, fluorescein angiograms showed no evidence of laser spots. No patients complained of ocular discomfort after SMDLP.

## 4. Discussion

The data from the present study demonstrate that SMDLP can effectively resolve macular edema and maintain visual acuity in Japanese patients with mild or moderate persistent macular edema secondary to BRVO, including patients with BCVA > 20/40. In the total subject-pool in this study, at 3 months after laser treatment, macular edema was significantly reduced (*P* = 0.014) and remained stable thereafter, and TMV was significantly reduced at 6 months (*P* = 0.002). Macular edema secondary to BRVO is typically self-resolving in nature [4, 25].

Hayreh and Zimmerman [25] reported median times to macular edema resolution of 21 months for major BRVO and 18 months for macular BRVO. It has been reported that macular edema due to BRVO naturally improves gradually over 18–21 months [25]. However, by design, all patients in the present study had persistent or recurrent macular edema, and the median time to progression to persistent macular edema was 34.6 months for BRVO, which was not indicative of a “self-resolving” condition. Therefore, the early response observed suggests that the observed resolution of macular edema was a direct effect of laser treatment, rather than spontaneous resolution. All patients in the present study had been followed for at least 6 months since disease onset, with 34.4% already having undergone treatment such as steroid [25, 26] or anti-VEGF therapy [2, 3]. Therefore, the resolution of edema documented in this study was apparently a direct result of laser therapy, rather than a natural course of healing.

Grid laser treatment has been a standard treatment for BRVO for many years. In 1984, the BVOS group reported that 65% of patients treated by argon laser grid photocoagulation gained more than 2 lines of visual acuity, compared with only 37% of eyes with untreated BRVO at 3 years follow-up [1]. Unfortunately, conventional grid laser treatment delivered with a visible ophthalmoscopic end-point has been implicated in several long-term complications that can severely affect visual function, including scar enlargement [6–8], subretinal fibrosis [27–30], choroidal neovascularization [31, 32], and perimetric sensitivity deterioration [33–38]. In an attempt to minimize the drawbacks of conventional grid laser photocoagulation, several authors have proposed the use of SMDLP for diabetic macular edema, which has shown promising results [14–21]. SMDLP involves the release of micropulses with low energy per pulse, in order to confine that energy to RPE cells, avoiding lateral thermal spreading. Histopathologically, retinal morphological changes are minimal and no immediate biomicroscopic retinal changes are noted after laser application, and there is no laser scarring on long-term follow-up [11]. Current spectral domain OCT scans cannot optimally discern laser ablation sites [13]. In the present study, no laser scars were detected in any patients after SMDLP.

The clinical application of SMDLP was first reported by Friberg and Karatza [14] in 1999. Since then, several clinical studies have demonstrated the efficacy of SMDLP [16–23]. In 2010, Ohkoshi and Yamaguchi [20] reported the efficacy of SMDLP for diabetic macular edema in Japanese patients. In 2010, Lavinsky et al. [21] reported that high density SMDLP was more effective than modified Early Treatment Diabetic Retinopathy Study (ETDRS) laser treatment, in a randomized clinical trial.

Although the efficacy of SMDLP has been proposed for diabetic macular edema, very few reports have been published for BRVO [22, 23]. In 2006, Parodi et al. [22] reported that the efficacy of SMDLP in macular edema due to BRVO with BCVA ≤ 20/40 was similar to that of conventional threshold grid laser treatment but without biomicroscopic or angiographic signs in the SMDLP group, at 12 months and 24 months. In that study however, macular edema was not significantly reduced at 3 months or 6 months.

In 2008, Parodi et al. [23] reported that they could not achieve significant reduction of macular edema due to BRVO with BCVA ≤ 20/40 within 6 months after SMDLP. In the present study, patients with BCVA ≤ 20/40 maintained BCVA and exhibited significant reductions in CMT at 6 months and 12 months. In this study, significant visual improvement was not achieved, although CMT was significantly improved at 6 months. This may be due to the fact that all of the patients had had chronic persistent macular edema for more than 6 months. Patients with early onset macular edema whose natural course of macular edema and BCVA would be self-improving were excluded from this study.

In this study, patients with BCVA > 20/40 maintained BCVA for 12 months and exhibited marginally significant reductions in CMT at 3 months and 12 months and a significant reduction at 6 months. Although most patients with good visual acuity such as BCVA > 20/40 were observed without any intervention in previous studies [1–5, 22, 23] and the results of this study suggest that early intervention with SMDLP may maintain BCVA and reduce macular edema in cases with good visual acuity.

## 5. Conclusions

In conclusion, in patients with persistent macular edema secondary to BRVO, SMDLP appears to control macular edema, with minimal retinal damage. Our findings suggest that SMDLP is an effective treatment method for macular edema in BRVO patients, including those with BCVA > 20/40. Limitations of this study include lack of randomization, the fact that it was not a prospective trial, and the relatively low number of patients. A randomized study would be necessary to prove the efficacy of SMDLP for macula edema secondary to BRVO.

## Figures and Tables

**Table 1:** Sample demographics.

Variable	Treatment group		*P* value
	Group I: eyes with BCVA ≤ 20/40	Group II: eyes with BCVA > 20/40	
Eyes, *n*	15	17	
Sex, *n* (%)			
Male	12 (80.0)	11 (64.7)	0.337
Female	3 (20.0)	6 (35.3)	
Age, mean (SD), years	70.53 (10.78)	63.65 (7.66)	0.044
Hypertension, *n* (%)	9 (60.0)	10 (58.8)	0.946
Cardiovascular disease, *n* (%)	5 (33.3)	1 (5.9)	0.047
Diabetes, *n* (%)	3 (20.0)	4 (23.5)	0.810
BRVO type			
Ischemic	7	6	0.513
Nonischemic	8	11	
Macular BRVO	7	7	0.755
Additional MP treatment, *n* (%)	8 (53.3)	3 (17.6)	0.034
Baseline BCVA (SD), log MAR	0.5933 (0.2277)	0.1263 (0.0769)	0.355
Baseline CMT (SD), *µ*m	409.266 (87.955)	373.294 (100.268)	0.756
Baseline TMV (SD), mm^3^	8.1273 (0.8761)	8.175 (2.8895)	0.952

BCVA: best-corrected visual acuity; BRVO: branch retinal vein occlusion; MP: micropulse photocoagulation; CMT: central macular thickness; TMV: total macular volume.

**Table 2:** Summary of data from subjects who did and did not undergo additional SMDLP.

Variable	Treatment group	
	No additional SMDLP	Additional SMDLP
Eyes, *n*	21	11
Sex, *n* (%)		
Male	17 (81.0)	7 (63.6)
Female	4 (19.0)	4 (36.4)
Age, mean (SD), years	68.48 (10.63)	63.82 (7.25)
BCVA (SD), log MAR		
Baseline	0.4062 (0.3071)	0.2297 (0.2108)
1 month	0.3599 (0.3262)	0.1839 (0.2255)
3 months	0.3687 (0.3101)	0.2281 (0.2308)
6 months	0.3777 (0.3788)	0.2132 (0.2255)
12 months	0.3476 (0.3535)	0.1939 (0.2390)
CMT (SD), *µ*m		
Baseline	393.524 (99.622)	383.727 (89.608)
1 month	369.809 (108.959)	377.909 (97.217)
3 months	313.857 (111.305)	391.091 (84.911)
6 months	267.095 (104.461)	372.000 (80.554)
12 months	266.240 (86.214)	400.182 (79.328)
TMV (SD), mm^3^		
Baseline	8.1168 (0.7531)	8.2109 (1.2705)
1 month	8.0005 (0.6299)	8.2080 (1.0580)
3 months	7.8533 (0.6394)	8.322 (1.0524)
6 months	7.49 (0.3861)	7.99 (0.8307)
12 months	7.5547 (0.4610)	7.9929 (0.5118)

BCVA: best-corrected visual acuity; BRVO: branch retinal vein occlusion; SMDLP: subthreshold micropulse diode laser photocoagulation; CMT: central macular thickness; TMV: total macular volume.

**Table 3:** Change in CMT and TMV after subthreshold micropulse diode laser photocoagulation.

	Baseline	1 month	3 months	6 months	12 months
Mean CMT (*μ*m) ± SD	390.16 ± 94.95	372.59 ± 103.55	340.41 ± 108.20	303.16 ± 108.15	312.28 ± 104.90
Mean TMV (mm^3^) ± SD	8.151 ± 0.954	8.072 ± 0.791	8.021 ± 0.867	7.657 ± 0.606	7.683 ± 0.508
Macular thickness reduction^a^ (%)		7/32 (21.9%)	12/32 (37.5%)	16/32 (50.0%)	14/32 (43.8%)

CMT: central macular thickness; TMV: total macular volume.

^a^Number of eyes with 20% or more reduction in CMT from baseline.

**Table 4:** Changes in BCVA after subthreshold micropulse diode laser photocoagulation (*n* = 32).

Change in BCVA^a^		1 month	3 months	6 months	12 months
Improved (*n*, %)	Total (*n* = 32)	3/32 (9.4%)	4/32 (12.5%)	3/32 (9.4%)	5/32 (15.6%)
	Group I (*n* = 15)	3/15 (20.0%)	4/15 (26.7%)	3/15 (20.0%)	5/15 (33.3%)
	Group II (*n* = 17)	0/17 (0%)	0/17 (0%)	0/17 (0%)	0/17 (0%)

Unchanged (*n*, %)	Total (*n* = 32)	29/32 (90.6%)	26/32 (81.3%)	26/32 (81.3%)	25/32 (78.1%)
	Group I (*n* = 15)	12/15 (80.0%)	11/15 (73.3%)	10/15 (66.7%)	9/15 (60.0%)
	Group II (*n* = 17)	17/17 (100.0%)	15/17 (88.2%)	16/17 (94.1%)	16/17 (94.1%)

Worsened (*n*, %)	Total (*n* = 32)	0/32 (0%)	2/32 (6.3%)	3/32 (9.4%)	2/32 (6.3%)
	Group I (*n* = 15)	0/15 (0%)	0/15 (0%)	2/15 (13.3%)	1/15 (6.7%)
	Group II (*n* = 17)	0/17 (0%)	2/17 (11.8%)	1/17 (5.9%)	1/17 (5.9%)

BCVA: best-corrected visual acuity; Group I: eyes with BCVA ≤20/40; Group II: eyes with BCVA > 20/40.

^a^Change in BCVA is defined as 0.2 or more log MAR (logarithm of the minimal angle of resolution) value. Improved: 0.2 or more log MAR gain; Unchanged: within 0.2 log MAR change; Worsened: 0.2 or more log MAR loss.
